# Preoperative pre-albumin predicts prognosis of patients after gastrectomy for adenocarcinoma of esophagogastric junction

**DOI:** 10.1186/s12957-016-1035-x

**Published:** 2016-11-03

**Authors:** Wen-xiu Han, Zhang-ming Chen, Zhi-jian Wei, A-man Xu

**Affiliations:** Department of Gastrointestinal Surgery, The First Affiliated Hospital of Anhui Medical University, Hefei, Anhui Province 230032 China

**Keywords:** AEG, Gastric cancer, Pre-albumin, PNI, OS

## Abstract

**Background:**

Adenocarcinoma of esophagogastric junction (AEG) was initially proposed in 1999 by Siewert. During recent decades, the incidence and prevalence of AEG were arising globally whereas the incidence of gastric cancer is gradually declining. Complete blood counting and liver function tests, as the routine examination of immune and nutritional status, were reported to be the predictors of overall survival (OS) in some tumors. However, little is known about the prognostic significance of these indexes in AEG patients. The purpose of this study was to assess the prediction of preoperative pre-albumin, hemoglobin, and prognostic nutritional index (PNI) for survival outcomes in AEG patients.

**Methods:**

A retrospective cohort of 101 AEG patients followed by radical surgery was recruited between January and July 2010. Clinical and laboratory data were obtained and used to evaluate the predictive value through survival analysis. Receiver operating characteristic (ROC) curve analysis determined 200 mg/L, 120 g/L, 5 cm, and 51 as the cutoff values of pre-albumin, hemoglobin, tumor size, and PNI, respectively.

**Results:**

Univariate analysis revealed that AEG patients with hemoglobin ≥120 g/L, albumin ≥40 g/L, pre-albumin ≥200 g/L, PNI ≥51, and tumor size <5 cm had longer OS (*P* < 0.05). Additionally, pre-albumin, tumor size, and TNM stage were demonstrated to be independent prognostic indicators by multivariate analysis with Cox regression, and the performance of pre-albumin for predicting OS in AEG patients was further identified by ROC curves (*P* = 0.006).

**Conclusions:**

Preoperative pre-albumin was an independent prognostic factor, and a high level of pre-albumin predicted longer OS in AEG patients.

## Background

Gastric cancer (GC) was one of the most prevalent malignant diseases worldwide and ranked second for cancer deaths in 2013, especially in developing countries [1]. Adenocarcinoma of esophagogastric junction (AEG), as one of special malignant tumors due to their borderline location between the esophagus and stomach, was initially proposed in 1999 by Siewert [2]. During recent decades, the incidence and prevalence of AEG were arising globally whereas the incidence of gastric cancer is gradually declining [3–5]. Siewert et al. proposed that tumors at the location within 5 cm from the Z-line were defined as AEG independently and classified as three types, which have been widely adopted worldwide [6]. Recent epidemiological and clinical studies suggested that the prevalence, etiology, pathology, treatments, and outcomes of AEG were distinguishing obviously from tumors at other locations, even in the three types of AEG [7].

The competition between tumor aggression and body defense is crucial for prognosis of cancer-related overall survivals (OS). Among them, immune and nutritional status of patients with cancer had gradually become the focus in the field of cancer research nowadays [8], especially in patients with postoperative chemotherapy. Complete blood counting and liver function tests, as the routine examinations before surgery, were reported to be the predictors of OS in some tumors, such colorectal cancer, breast cancer, hepatocellular carcinoma, and GC [8–11]. Hemoglobin and albumin were the most common parameters to reflect the nutritional status, which of the lower level was demonstrated to be associated with poorer prognosis for patients with GC. Additionally, neutrophils to lymphocytes ratio (NLR) and platelets to lymphocytes ratio (PLR), as predictors of patients with GC, have been studied worldwide.

To our best knowledge, no studies had republished to access the prediction of these indexes for survival outcome in patients with AEG who underwent gastrectomy and chemotherapy. Here, the aim of this study was to research the clinical values of these parameters for prediction of OS in AEG patients.

## Methods

### Patients

The patients, who underwent radical open total or proximal gastrectomy for primary gastric cancer and were diagnosed as adenocarcinoma of esophagogastric junction based on postoperative pathology in the First Affiliated Hospital of Anhui Medical University (FAHAMU) from January to July 2010, were enrolled in this study retrospectively. All patients received the preoperative examinations of hemoglobin, albumin, pre-albumin, neutrophil, lymphocyte, and platelet. Additionally, complete clinicopathological characteristics including age, gender, tumor site, differentiation grade, tumor size, infiltration depth, lymph node metastasis, and distant metastasis were also collected. Patients who died within 30 days after surgery and received preoperative chemoradiotherapy were excluded from this study. Patients were also excluded if they underwent splenectomy or hepatectomy and had evidence of infections or were diagnosed with autoimmune diseases and multiple primary cancers. Finally, a total of 101 patients, who were followed up through telephones and outpatient visit up to September 2015, were enrolled in this study.

### Clinical and laboratory data collection

All the details were collected from FAHAMU cancer database. The blood samples were gathered within 7 days before surgery to examine the hemoglobin, neutrophil, lymphocyte, platelet, albumin, globulin, and pre-albumin. According to the seventh edition of the American Joint Committee on Cancer (AJCC 2010) on tumor-node-metastasis (TNM) staging [12], the postoperative pathological stages were determined for patients with gastric cancer. Additionally, all AEG patients were classified into three types based on the AEG criteria recommended by Siewert (1998).

### Definition of prognostic nutritional index and cutoff values

The prognostic nutritional index (PNI) was calculated using the following formula: 10 × serum albumin (g/dL) + 0.005 × total lymphocyte count (per mm^3^) [13]. The PNI cutoff points were selected by receiver operating characteristic (ROC) curve analysis for the prediction of survival outcomes based on data from the whole cohort. Finally, using the Youden index [maximum (sensitivity + specificity − 1)] [14], we determined that the recommended cutoff value was 51 [sensitivity, 69.2; specificity, 59.2; area under the curve (AUC), 0.615; *P* = 0.046]. And according to the recommended cutoff value, patients were divided into groups as follows: low-PNI group (PNI <51) and high-PNI group (PNI ≥51).

The recommended cutoff values for preoperative hemoglobin and pre-albumin were decided using ROC curve analysis based on the most prominent points on the ROC curves and defined as 120 g/L [sensitivity, 51.9; specificity, 71.4; area under the curve (AUC), 0.621; *P* = 0.037] and 200 mg/L [sensitivity, 44.0; specificity, 88.0; area under the curve (AUC), 0.659; *P* = 0.006].

### Statistical analysis

Statistical Package for the Social Sciences, version 21.0 (SPSS, IBM, Chicago, IL, USA), was used for all statistical analysis, and differences at *P* value <0.05 were considered to be significant in all statistical analysis. The ROC curves were constructed to determine the cutoff values of hemoglobin, pre-albumin, PNI, and tumor size. Additionally, the relationships between associated factors and overall survival were analyzed through the Kaplan-Meier method and compared by the log-rank test, respectively. Moreover, multivariate analysis was performed based on the univariate analysis with *P* < 0.05 to evaluate the most valuable predictor of survival outcomes.

## Results

### Baseline of patients’ characteristics

The clinicopathological characteristics of the 101 AEG patients and their relationships with overall survival were summarized in Table 1. Overall, 80 (79.2 %) patients were males and 21 (20.8 %) were females. The mean age of patients was 65 years old (range, 43–82). The median follow-up period was 51 months (range, 1.5–66), and there were 52 (51.5%) cases confirmed as dead at the last follow-up.

**Table 1:** Clinical and laboratory characteristics of 101 AEG patients associated with OS

Patient-related factors	No. of patients (%)	OS (months) [mean (95 % CI)]	*P* values
Gender			
Male	80 (79.2)	42.3 (36.9–47.7)	0.343
Female	21 (20.8)	48.5 (39.4–57.7)	
Age (years)			
<60	23 (22.8)	44.5 (34.9–54.1)	0.976
≥60	78 (77.2)	43.3 (38.9–48.3)	
BMI (kg/m^2^)			
<18.5	9 (8.9)	31.7 (18.8–14.6)	0.180
≥18.5 and <25	71 (70.3)	45.5 (40.0–51.1)	
≥25	21 (20.8)	41.4 (30.7–52.0)	
Hemoglobin (g/L)			
<120	41 (40.6)	38.4 (31.4–45.4)	0.029*
≥120	60 (59.4)	44.6 (41.0–53.4)	
Albumin (g/L)			
<40	27 (26.7)	35.7 (26.4–44.9)	0.036*
≥40	74 (73.3)	46.0 (38.9–48.3)	
Pre-albumin (g/L)			
<200	29 (28.7)	31.5 (23.6–39.3)	<0.001*
≥200	72 (71.3)	48.0 (43.0–53.8)	
PNI			
<51	54 (53.5)	37.6 (31.0–44.2)	0.008*
≥51	47 (46.5)	50.5 (44.3–56.7)	
Tumor size (cm)			0.004*
<5	64 (63.4)	48.3 (42.5–54.1)	
≥5	37 (36.6)	35.4 (28.0–42.8)	
Differentiation grade			
Poor	69 (68.3)	39.4 (33.5–45.3)	0.031*
Well	32 (31.7)	52.0 (45.0–58.0)	
Tumor location			
Siewert II	6 (5.9)	40.3 (19.6–61.1)	0.923
Siewert III	95 (5.1)	43.8 (38.9–48.6)	
T stage			
T1, T2	18 (17.8)	61.9 (56.2–67.6)	0.001*
T3, T4	83 (82.2)	39.6 (34.4–44.9)	
N stage			
N0	41 (40.6)	51.1 (43.9–58.4)	0.001*
N1	43 (42.6)	41.3 (34.7–47.9)	
N2	16 (15.8)	32.5 (20.8–44.2)	
N3	1 (1.0)	8.0 (8.0–8.0)	
TNM stage			
I, II	44 (43.6)	52.5 (45.7–59.2)	<0.001*
III, IV	57 (56.4)	36.7 (30.8–42.7)	
Surgical type			
Total gastrectomy	6 (5.9)	57.0 (42.7–71.3)	0.131
Proximal gastrectomy	95 (94.1)	42.7 (37.8–47.6)	

*BMI* body mass index, *PNI* prognostic nutritional index

*P < 0.05

### Univariate analysis of prognostic factors

**Fig. 1:**
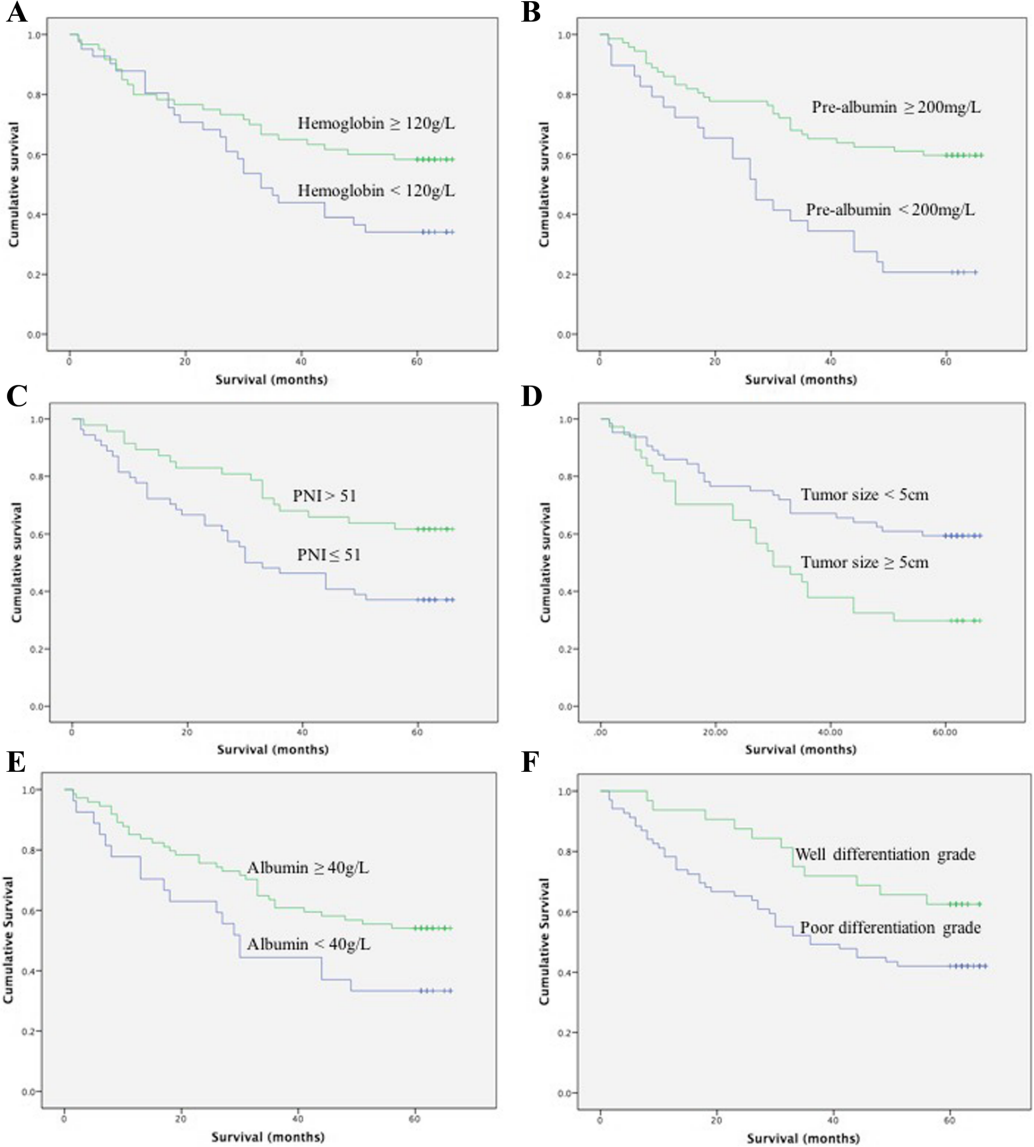
Kaplan-Meier survival curves after surgery for AEG patients in high hemoglobin and low hemoglobin (**a**), high pre-albumin and low pre-albumin (**b**), high prognostic nutritional index (PNI) and low PNI (**c**), large tumor size and small tumor size (**d**), high albumin and low albumin (**e**), and well differentiation grade and poor differentiation grade (**f**)

Among all 101 AEG patients, there are no significant differences observed in gender, age, BMI, and tumor location associated with OS. However, according to the univariate analysis, AEG patients with hemoglobin ≥120 g/L, albumin ≥40 g/L, pre-albumin ≥200 g/L, tumor size <5 cm, PNI ≥51, and well differentiation grade had longer OS (*P* < 0.05), and this was consistent with an earlier T stage, N stage, or TNM stage (*P* < 0.01). Moreover, larger tumor size was a significant risk factor for survival outcomes (*P* < 0.01) (Fig. 1).

### Multivariate analysis of prognostic factors

Multivariate analysis with Cox regression was performed to assess for various prognostic factors. Consistent with univariate analysis, pre-albumin [hazard ratio (HR) 0.512; 95 % CI 0.282–0.927; *P* = 0.027] and TNM stage (HR 2.532; 95 % CI 1.220–5.523; *P* = 0.013) were independent prognostic indicators and a high level of pre-albumin demonstrated a positive survival. However, the level of hemoglobin, tumor size, and PNI were not a significant independent factor in multivariate analysis (*P* > 0.05) (Table 2).

**Table 2:** Multivariate analysis of prognostic factors for OS in AEG patients

Patient-related factors	Hazard ratio	95 % CI	*P* value
Albumin (g/L)			
<40	1		0.874
≥40	0.945	0.469–1.903	
Hemoglobin (g/L)			
<120	1		1.000
≥120	1.000	0.527–1.899	
PNI			
<51	1		0.426
≥51	0.751	0.372–1.518	
Pre-albumin (g/L)			
<200	1		0.021*
≥200	0.494	0.271–0.901	
Tumor size (cm)			
<5	1		0.869
≥5	1.053	0.567–1.957	
TNM stage			
I, II	1		0.013*
III, IV	2.530	1.220–5.248	
Differentiation grade			
Poor	1		0.248
Well	1.335	0.307–1.357	

*PNI* prognostic nutritional index

*P < 0.05

### Definition of prognostic factors

**Fig. 2:**
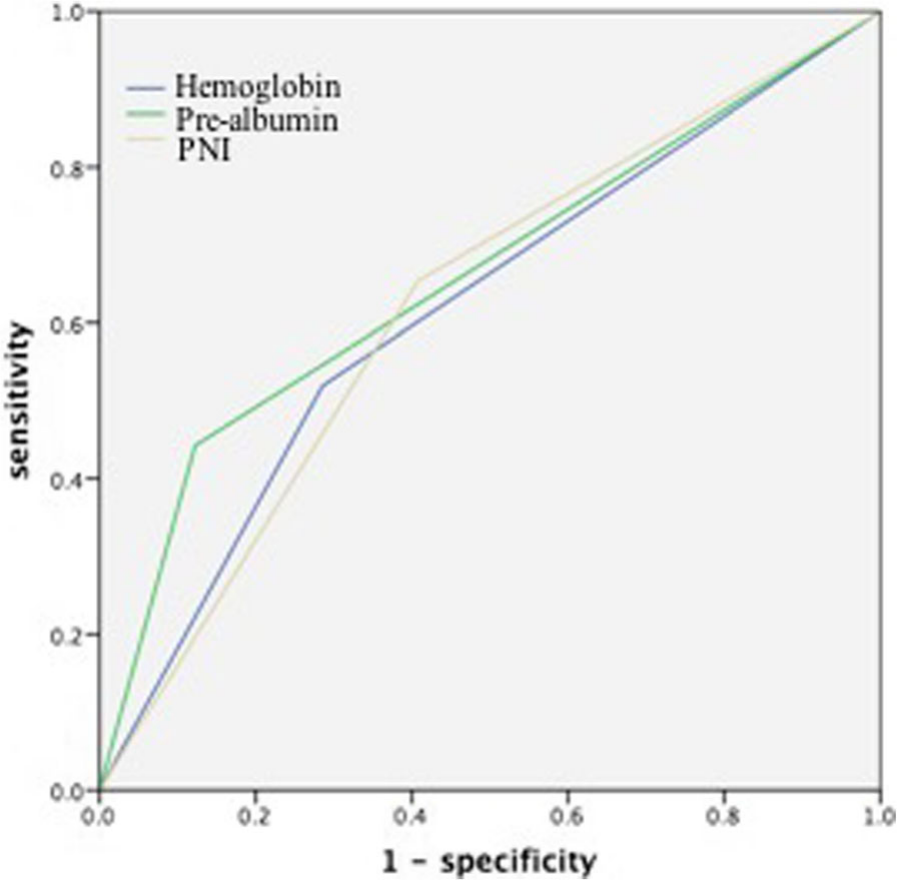
The predictive ability of the three parameters for 5-year OS was compared by ROC curves (PNI represents prognostic nutritional index)

Considering the interactions of patient-related factors for survival outcomes, ROC curves were constructed to estimate their discrimination ability (Fig. 2). The hemoglobin AUC was 0.617 (95 % CI 0.507–0.727); the pre-albumin AUC was 0.660 (95 % CI 0.553–0.767); and the PNI AUC was 0.623 (95 % CI 0.513–0.733). Therefore, the performance abilities of pre-albumin were similar to others for predicting overall survivals of AEG patients.

### Relationship between the pre-albumin and clinicopathologic characteristics

The serum pre-albumin, as an independent prognostic indicator, was associated with survival outcomes in AEG patients. Thus, subgroup analyses were further established to identify the relationships between the pre-albumin and other clinicopathologic characteristics and evaluate the prognostic value of pre-albumin more comprehensively (Table 3). Among them, a total of 29 AEG patients were detected with a lower level of pre-albumin <200 mg/L, whereas others were detected with a higher level of pre-albumin ≥200 mg/L. The pre-albumin was not significantly correlated with gender, age, BMI, albumin, and tumor location. However, the associations between pre-albumin and hemoglobin (*P* = 0.014), pre-albumin and PNI (*P* = 0.011), and pre-albumin and differentiation grade (*P* = 0.011) were significant. More importantly, the level of serum pre-albumin was significantly associated with TNM stage (*P* < 0.001).

**Table 3:** Relationship between the pre-albumin and clinicopathologic characteristics

Patient-related factors	Pre-albumin <200 mg/L	Pre-albumin ≥200 mg/L	*P* value
	(*n* = 29)	(*n* = 72)	
Gender			0.260
Male	22	58	
Female	7	14	
Age (years)			0.565
<60	4	19	
≥60	25	53	
BMI (kg/m^2^)			0.084
<18.5	7	2	
18.5 ≤ < 23	13	38	
23≤	9	32	
Hemoglobin (g/L)			0.014*
<120	17	24	
≥120	12	48	
Albumin (g/L)			0.052
<40	14	13	
≥40	15	59	
PNI			0.011*
<51	20	34	
≥51	9	38	
Differentiation grade			0.044*
Poor	20	49	
Well	9	23	
Tumor location			0.632
Siewert II	1	5	
Siewert III	28	67	
Tumor size (cm)			0.004*
<5	14	50	
≥5	15	22	
TNM stage			<0.001*
I, II	10	34	
III, IV	19	38	

*BMI* body mass index, *PNI* prognostic nutritional index

*P < 0.05

## Discussion

In the few decades, there has been an alarming rise in the incidence of tumors originating at the esophagogastric junction. In some literature, some authors held the opinion that all tumors arising at or close to the esophagogastric junction should be traditionally classified into esophageal carcinomas or gastric cancers, while Siewert et al. considered them as an entity called AEG and classified them into three types: I–III [2, 15–17]. In Eastern countries, types I and II of AEG were more prevalent than type III, which is in sharp contrast with the prevalence of the three types in Western countries [18]. The AEG-special etiological factors for dramatic increase of prevalence were remaining not determined. Continuous gastroesophageal reflux was reported to increase the risk of epithelium to progress to Barrett’s esophagus or adenocarcinoma [19]. Complete removal of primary tumor (R0 resection) with lymphadenectomy (D2), as routine radical surgery, remains the curative treatment that provided best survival outcomes. Nowadays, the differences of the 5-year survival rates (>50 %) were demonstrated to having no obvious significance in subtypes and better than tumors at other locations of GC, which was consistent with the results of this study [20].

Preoperative nutritional status is one of critical factors for patient outcomes in a variety of surgeries, especially in gastrectomy. Currently, pre-albumin became the research focus as a serum biomarker for assessment of nutritional status due to shorter half-life (about 1.9 days) than albumin, which is a negative acute-phase protein synthesized in the liver. Additionally, because of the intense from gastric cancer surgery, acute-phase proteins were synthesized in the liver from structural proteins in the plasma. Therefore, the level of pre-albumin has a high sensitivity for understanding the metabolism state and immunity of the body. This study found that a high level of pre-albumin predicted a longer OS than low level in AEG patients. Moreover, pre-albumin was an independent factor for predicting postoperative survival outcomes. Li et al. reported that postoperative levels of pre-albumin and high-sensitivity C-reactive protein were associated with short-term outcomes and complications after gastrectomy, especially in elderly patients [21]. Due to absence of complete postoperative pre-albumin for this study, the relationships with surgical intense and short-term outcomes had not been determined further in AEG patients.

Another index for assessing the immune and nutrition status was PNI, which was demonstrated to predicting the survival outcomes in patients after gastric cancer surgery. This study suggested AEG patients with a high level of PNI had a longer OS than others, although it was not an independent predictor in univariate analysis. One study of 548 patients with gastric cancer who underwent gastrectomy found that low PNI was associated with less tumor depth and lymph node metastasis [22]. Currently, the cutoff values of PNI in different studies were not same which were determined by ROC curves or X-title program, so a deterministic value of PNI to assessing survival outcomes is not scientific.

The major limitations of this study were lack of complete specific tumor markers and inflammatory markers and was a retrospective single-center clinical research. Because patients with AEG of type I were treated by the Department of Thoracic Surgery, the subtype analysis had not been completed.

## Conclusions

This study first established the relationships between preoperative indexes of hematology and prognosis of AEG patients and found that AEG patients with a high level of pre-albumin, hemoglobin, and PNI had longer OS. Moreover, our study further demonstrated that preoperative pre-albumin was an independent prognostic factor.

## Abbreviations

AEG: Adenocarcinoma of esophagogastric junction
AJCC: American Joint Committee on Cancer
FAHAMU: First Affiliated Hospital of Anhui Medical University
NLR: Neutrophil to lymphocytes ratio
OS: Overall survival
PNI: Prognostic nutritional index
ROC: Receiver operating characteristic
SPSS: Statistical Package for the Social Sciences
TNM: tumor-node-metastasis
